# Clinical commissioning of a new patient positioning system, SyncTraX FX4, for intracranial stereotactic radiotherapy

**DOI:** 10.1002/acm2.12467

**Published:** 2018-10-01

**Authors:** Satoshi Tanabe, Osamu Umetsu, Toshikazu Sasage, Satoru Utsunomiya, Ryota Kuwabara, Toshiki Kuribayashi, Hiromasa Takatou, Gen Kawaguchi, Hidefumi Aoyama

**Affiliations:** ^1^ Department of Radiation Oncology Niigata University Medical and Dental Hospital Niigata Japan; ^2^ Department of Radiation Oncology Uonuma Kikan Hospital Niigata Japan; ^3^ Department of Radiological Technology Niigata University Graduate School of Health Sciences Niigata Japan; ^4^ Department of Radiology and Radiation Oncology Niigata University Graduate School of Medical and Dental Sciences Niigata Japan

**Keywords:** commissioning, image‐guided radiotherapy (IGRT), stereotactic radiotherapy

## Abstract

**Background & Aims:**

A new real‐time tracking radiotherapy (RTRT) system, the SyncTraX FX4 (Shimadzu, Kyoto, Japan), consisting of four X‐ray tubes and four ceiling‐mounted flat panel detectors (FPDs) combined with a linear accelerator, was installed at Uonuma Kikan Hospital (Niigata, Japan) for the first time worldwide. In addition to RTRT, the SyncTraX FX4 system enables bony structure‐based patient verification. Here we provide the first report of this system's clinical commissioning for intracranial stereotactic radiotherapy (SRT).

**Materials & Methods:**

A total of five tests were performed for the commissioning: evaluations of (1) the system's image quality; (2) the imaging and treatment coordinate coincidence; and (3) the localization accuracy of cone‐beam computed tomography (CBCT) and SyncTraX FX4; (4) the measurement of air kerma; (5) an end‐to‐end test.

**Results & Discussion:**

The tests revealed the following. (1) All image quality evaluation items satisfied each acceptable criterion in all FPDs. (2) The maximum offsets among the centers were ≤0.40 mm in all combinations of the FPD and X‐ray tubes (preset). (3) The isocenter localization discrepancies between CBCT and preset #3 in the SyncTraX FX4 system were 0.29 ± 0.084 mm for anterior‐posterior, −0.19 ± 0.13 mm for superior‐inferior, 0.076 ± 0.11 mm for left‐right, −0.11 ± 0.066° for rotation, −0.14 ± 0.064° for pitch, and 0.072±0.058° for roll direction. the Pearson's product‐moment correlation coefficient between the two systems was >0.98 in all directions. (4) The mean air kerma value for preset #3 was 0.11 ± 0.0002 mGy in predefined settings (80 kV, 200 mA, 50 msec). (5) For 16 combinations of gantry and couch angles, median offset value in all presets was 0.31 mm (range 0.14–0.57 mm).

**Conclusion:**

Our results demonstrate a competent performance of the SyncTraX FX4 system in terms of the localization accuracy for intracranial SRT.

## 1 INTRODUCTION

Image‐guided radiotherapy (IGRT) is becoming crucial for further innovation in conformal radiotherapy, as the use of IGRT ensures that high‐precision techniques are delivered as planned.^1^ In particular, high localization accuracy (typically within 1 mm) is needed in intracranial stereotactic radiotherapy (SRT) in order to not compromise the local control and to minimize the risk of intracranial complications.^2^ Several research groups reported that IGRT techniques including orthogonal kV‐imaging,^3^ oblique kV‐imaging,^4^, ^5^, ^6^, ^7^, ^8^, ^9^ kV‐cone‐beam computed tomography (CBCT),^2^, ^5^, ^9^, ^10^, ^11^, ^12^, ^13^ and megavoltage (MV)‐CT^14^, ^15^ have high accuracy for positioning verification in intracranial SRT. However, a clinical problem is presented by intracranial SRT plans that consist of a number of noncoplanar beams that cannot be achieved with the use of the above‐cited techniques.

**Figure 1:**
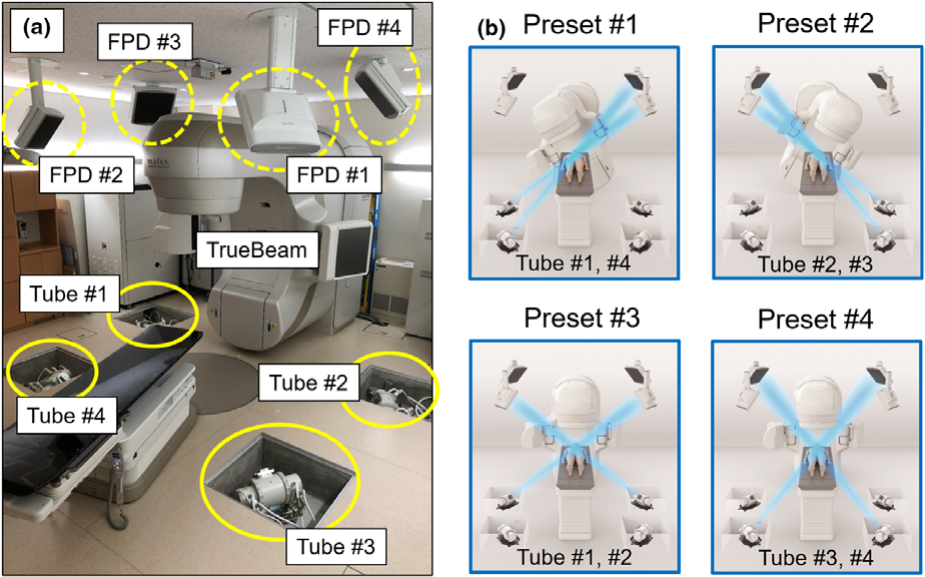
(a) An overview of SyncTraX FX4 system with the TrueBeam. (b) Four predetermined combinations of x‐ray tubes and flat panel detectors (presets).

At Uonuma Kikan Hospital (Niigata, Japan), a new real‐time tracking radiotherapy (RTRT) system, the SyncTraX FX4 (Shimadzu Co., Kyoto, Japan), was installed for the first time in the world. The SyncTraX FX4 system consists of four x‐ray tubes and four ceiling‐mounted flat panel detectors (FPDs) combined with a linear accelerator (TrueBeam; Varian Medical Systems, Palo Alto, CA), as shown in Fig. 1(a). The principle of this system for RTRT is similar to the previous RTRT systems described by Shirato et al.^16^ and Shiinoki et al.^17^ However, the SyncTraX FX4 differs from the previous systems in two notable ways.^16^, ^17^

**Figure 2:**
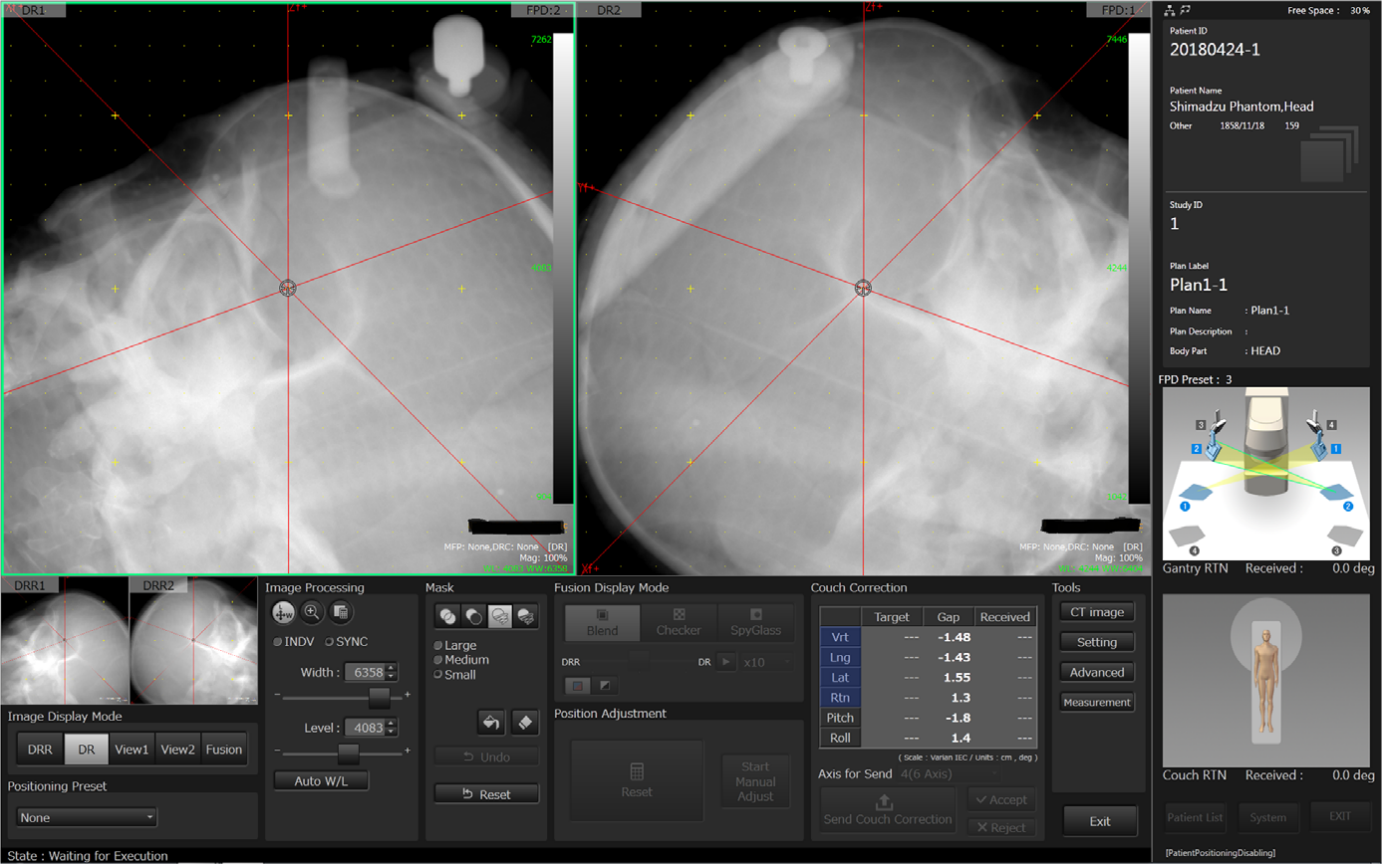
Verification image of SyncTraX FX4‐based bone matching using a head phantom.

First, these systems’ detectors differ; that is, an image intensifier (I.I.) is used in the previous systems, whereas FPDs are used in the SyncTraX FX4 system. The influence of image distortion caused by the use of an I.I. has thus been eliminated, and bony structure‐based verification became possible with the SyncTraX FX4 as a patient verification system (Fig. 2). Second, the designs of the x‐ray tube and the detector are different. As shown in Fig. 1(b), the imaging positions of the x‐ray tubes and FPDs of the SyncTraX FX4 can be selected from a total of four combinations called “presets”. In addition, since the configuration of the x‐ray tubes in the SyncTraX FX4 system has changed from the previous rail‐type system to the fixed type, it is possible to switch the imaging direction promptly compared to the previous systems. Thus, since there are very few blind angles with regard to the gantry and couch angles of the SyncTraX FX4, this system could be assumed to be effective for intracranial SRT. However, there is no report about positioning verification for radiotherapy using the SyncTraX FX4. We conducted the present study to provide a first report of the clinical commissioning of a SyncTraX FX4 system for intracranial SRT.

## 2 MATERIALS AND METHODS

### 2.A Image quality

**Figure 3:**
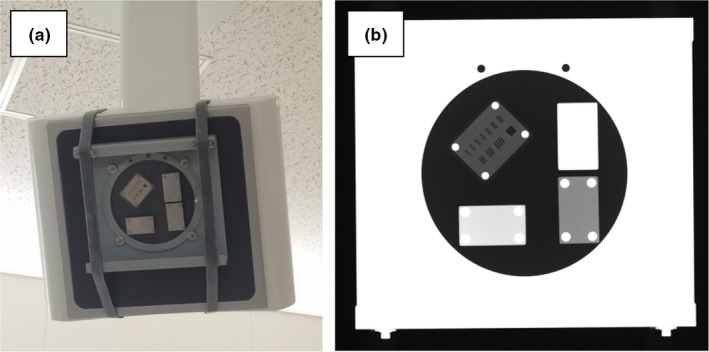
(a) Image evaluation phantom. (b) Image of the image evaluation phantom taken under the following conditions: 80 kV, 250 mA, 10 ms.

For the evaluation of the image quality of each FPD in the SyncTraX FX4 system, we used an image evaluation phantom (Shimadzu). This phantom was composed of aluminum plates with different resolution and thickness values, and it was attached in front of an FPD before measurement, as shown in Fig. 3(a). Images were taken and acquired under the following conditions: 80 kV, 250 mA, 10 ms [Fig. 3(b)]. Based on the American Association of Physicists in Medicine (AAPM) task group (TG)‐142 guideline,^18^ we examined four evaluation items: scaling, spatial resolution, contrast resolution, and uniformity.

**Figure 4:**
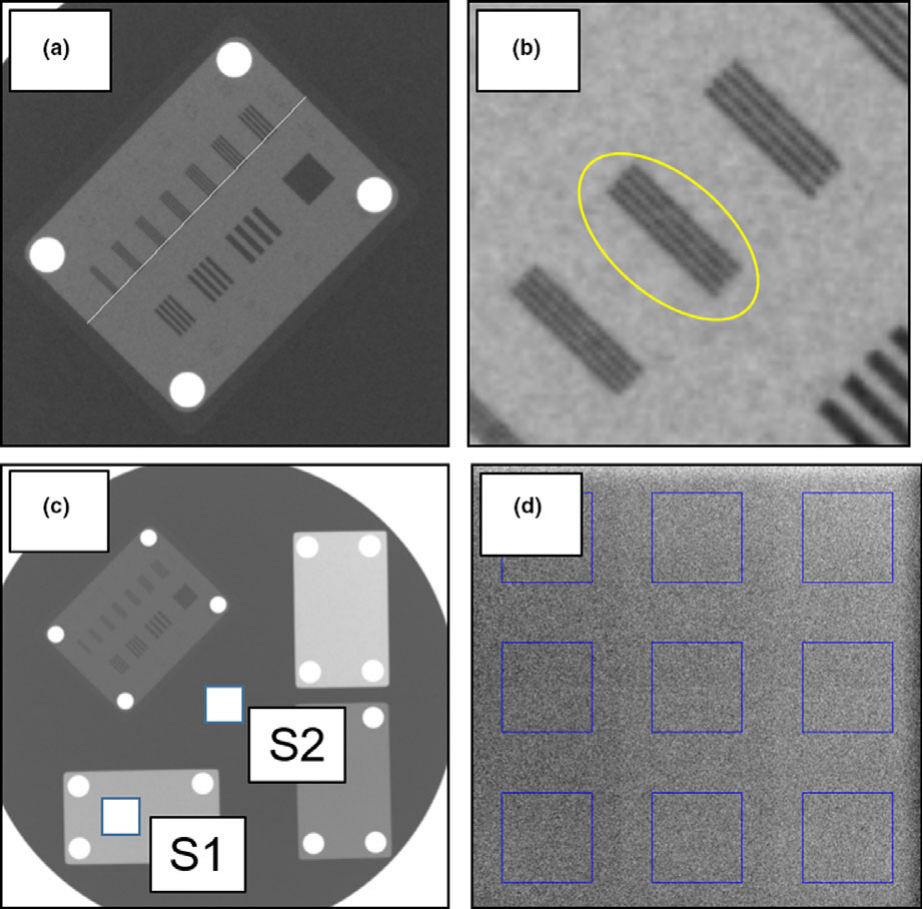
Image of the image evaluation phantom. (a) Scaling. (b) Spatial resolution. (c) Contrast. (d) Uniformity.

Scaling was evaluated by measuring the long side of the resolution chart [Fig. 4(a)]. Spatial resolution was assessed by visually determining each line pair (lp) [Fig. 4(b)]. For contrast resolution, we calculated the contrast‐to‐noise ratio (CNR) using the following formula:

(1) $$\text{CNR}=\frac{|S1_{\mathrm{mean}}-S2_{\mathrm{mean}}|}{S2_{\mathrm{SD}}}$$

where *S*1_*mean*_ and *S*2_*mean*_ are the mean pixel values over a region‐of‐interest (ROI) in an aluminum plate [Fig. 4(c), “S1”) and in the background regions [Fig. 4(c), “S2”], respectively, and *S*2_*SD*_ is the standard deviation of the pixel values in the ROI in the background region.

For the evaluation of uniformity, we divided the images taken without attaching the phantom into nine ROIs [Fig. 4(d)] and calculated the coefficient of variation (CV) using the following formula:

(2) $$\text{CV}=\frac{SD_{M}}{Mean_{M}}\times 100$$

where *SD*_*M*_ is the mean value of the standard deviation for each pixel and *Mean*_*M*_ is the mean value of the average for each pixel. We set the acceptance criteria as a measurement error <0.5 mm for scaling, a CNR >50 for contrast resolution, and a CV <5% for uniformity.

### 2.B Imaging and treatment coordinate coincidence

**Figure 5:**
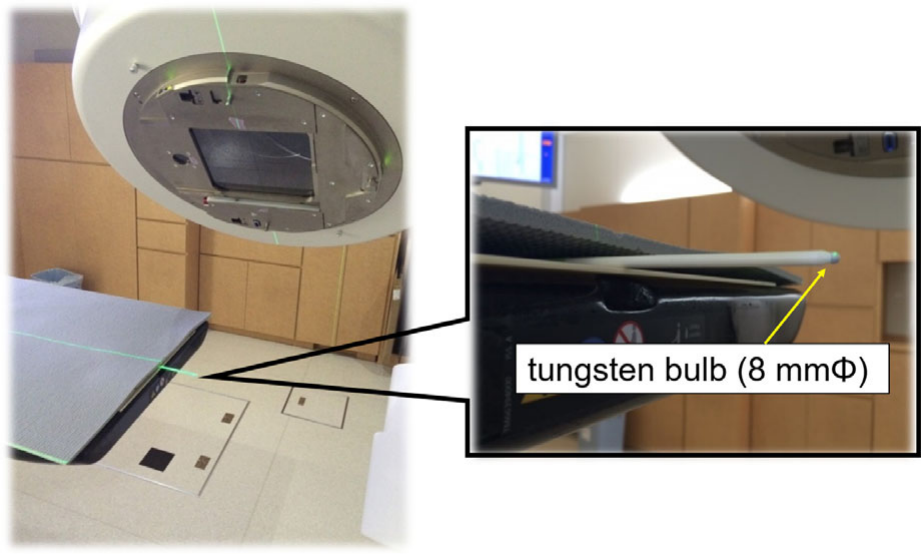
Measurement setup to confirm the degree of coordinate coincidence of the isocenter for each coordinate system. The center of a tungsten ball was aligned with the radiation isocenter of the TrueBeam.

We evaluated the coincidence between the imaging coordinate and that of the treatment systems based on the TG‐142 report. The spatial displacement between the radiation center in the TrueBeam and the image center in the SyncTraX FX4 was measured using a phantom with a tungsten sphere (Fig. 5). The tungsten sphere was placed at the radiation center using RIT 113 ver. 6.3 software (Radiological Imaging Technology, Colorado Springs, CO).

We performed the measurements at gantry angles of 0°, 90°, 180°, and 270° so that the error of each axis was within 0.05 mm. After acquiring images for each of the four presets with the SyncTraX FX4, we measured the discrepancy between the radiation center and the image center in pixel units using ImageJ software (U.S. National Institutes of Health, Bethesda, MD). We then calculated the amount of discrepancy in the anterior–posterior (AP) axis (i.e., the positive direction corresponds to the anterior), the superior–inferior (SI) axis (the positive direction corresponds to the superior), and the left–right (LR) axis (the positive direction corresponds to the left) to convert from pixel unit to mm units. We also evaluated the coordinate coincidence between the kV‐planar images obtained with an on‐board‐imager (OBI) and those obtained with the SyncTraX FX4.

### 2.C Comparison of the localization accuracies of CBCT and SyncTraX FX4

**Figure 6:**
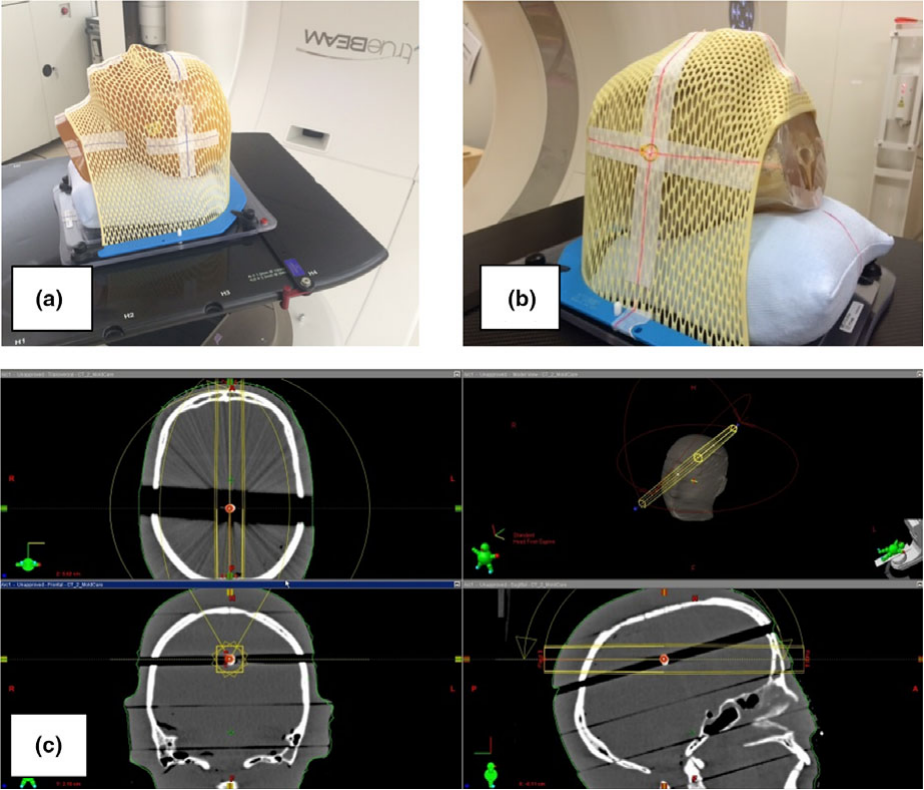
(a) Head phantom used to evaluate the localization accuracy between CBCT and the SyncTraX FX4. (b) Head phantom with a 5‐mm‐dia. gold marker used for the end‐to‐end test. (c) A treatment planning CT image for the end‐to‐end test. The isocenter in the treatment planning was aligned with the center of the gold marker.

We used the head phantom shown in Fig. 6(a) to determine and compare the localization accuracy of CBCT and the SyncTraX FX4. After we shaped a commercial thermoplastic mask (Qfix; Avondale, PA) to immobilize the head phantom, images were taken at a slice thickness of 1 mm using a CT scanner (SOMATOM Definition AS, Siemens, Erlangen, Germany). The CT isocenter was set at the center of the skull base. Next, the CT dataset was taken into the treatment planning device Eclipse ver. 13 (Varian Medical Systems), and the planning‐isocenter for the treatment plan was also set to the center of the skull base.

For the positional verification, we used skin marker‐based matching to place the phantom at the planning isocenter. The couch was randomly moved within a range of 0–20 mm for translational shifts (AP, SI, LR) and within a range of 0°–1.5° for rotational shifts around the AP axis (yaw), the SI axis (roll), and the LR axis (pitch). We then carried out bony structure (BS)‐based matching using CBCT to correct the setup errors through automatic image registration. Subsequently, BS‐based matching was performed automatically using the SyncTraX FX4. A total of 20 datasets of setup errors were acquired for each preset. We examined the correlation of the shifts between the CBCT and SyncTraX FX4 system.

We determined the Pearson's correlation coefficient between the CBCT and SyncTraX FX4 system relative to the position of the skin marker‐based matching. We then plotted the differences between each shift against the average shift by performing a Bland‐Altman analysis to assess the fixed bias. The average value, standard deviation, and root‐mean‐square (RMS) of the difference between each shift were calculated for each preset.

### 2.D Radiation dose of the SyncTraX FX4

**Figure 7:**
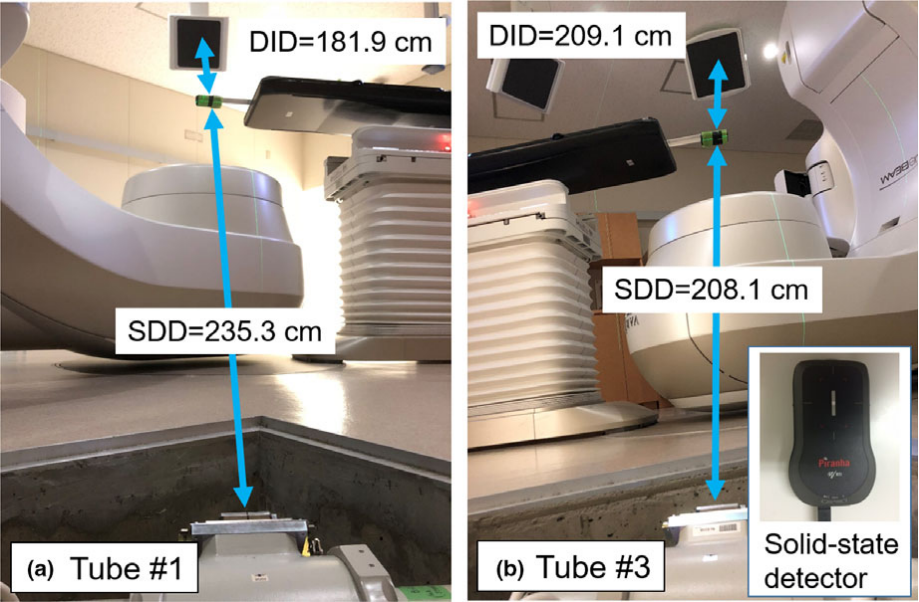
Experimental setup for the measurement of air kerma in (a) Tube #1 and (b) Tube #3. DID and SDD represent the detector‐to‐imager distance and source‐to‐detector distance respectively.

With the SyncTraX FX4 system, imaging x‐rays can be delivered at an angle of 37.7° from two x‐ray tubes (#1 and #2) with a source‐to‐detector distance (SDD) value of 235.3 cm and a detector‐to‐imager distance (DID) value of 181.9 cm, and at an angle of 43.8° from the other two tubes (#3 and #4) with the SDD value of 208.1 cm and the DID value of 209.1 cm (Fig. 7). Tube #1 and #2 were farthest from the isocenter and was observed to provide the minimum imaging radiation dose. Tube #3 and #4 were the nearest to the isocenter; it provided the maximum radiation dose. In order to evaluate the radiation doses of presets #3 and #4, we measured the air kerma at the isocenter of each exposure from Tube #1 [Fig. 7(a)] and #3 [Fig. 7(b)] with a solid‐state dosimeter (Piranha; RTI Electronics, Anaheim, CA) for several predetermined imaging parameters settings.

The detector was placed orthogonal to the beam from the x‐ray tube. At irradiation, the measurements were taken with one side of the x‐ray tube shielded with lead. The tube current was set at 50, 80, 100, 200, 250, 320, 400, and 500 mA, and the tube voltage was set at 70, 80, 90, 100, and 110 kV, and the exposure time was set at 50 ms. The measurement was carried out five times in each of the 40 imaging conditions, and the average value and the standard deviation were calculated. In the present study, under the assumption that the radiation dose of two tubes is equal for each of the four presets, twice the average value was taken as the air kerma per one measurement.

### 2.E End‐to‐end test

As shown in Fig. 6(b), a commercial thermoplastic mask (Qfix) was shaped for a head phantom with a 5‐mm‐dia. gold marker embedded. Planning CT images were then obtained at a slice thickness of 1 mm using a CT scanner (SOMATOM Definition AS). The isocenter on the treatment planning was set at the marker center, as shown in Fig. 6(c). After we created the digitally reconstructed radiography (DRR) on the Eclipse system, it was transferred to the TrueBeam and SyncTraX FX4 systems respectively.

For the position verification, we selected a BS‐based setup using the SyncTraX FX4 so that the image‐center of the SyncTraX FX4 system was consistent with that of the gold marker. Thereafter, the MV x‐ray beam was delivered to 2 × 2 cm^2^ fields to electronic portal imaging device (EPID) under the conditions of gantry angles of 0°, 135°, 180°, and 315° and couch angles of 0°, 30°, 60° and 90°. The offset between the center of the marker and the image center was evaluated by ImageJ software in the same manner as described in Section 2.B (Imaging and treatment coordinate coincidence).

## 3 RESULTS

### 3.A Image quality

The discrepancy between the actual and measured distances for FPD #3 was within 0.5 mm. For the spatial resolution, a chart of 2.3 lp/mm was identified. The CNR for contrast resolution was 101.9, and the CV for uniformity was 0.16%. The image quality results of the other three FPDs satisfied each of the four criteria, as shown in Table 1.

**Table 1:** Evaluation of each item for image quality for each FPD (flat panel detector)

	Scaling distance (mm)	Spatial resolution	Contrast CNR	Uniformity CV (%)
FPD #1	50.09	Identifiable	110.9	0.23
FPD #2	50.23	Identifiable	104.9	0.10
FPD #3	50.23	Identifiable	101.9	0.16
FPD #4	50.22	Identifiable	102.4	0.11

CNR: contrast‐to‐noise ratio; CV: coefficient of variation.

### 3.B Imaging and treatment coordinate coincidence

Table 2 summarizes the discrepancy of the coordinates between the image/radiation centers of the Truebeam and the image center of the SyncTraX FX4. In all four presets, the discrepancies between the radiation center of the Truebeam and the image center of the SyncTraX FX4 were <0.4 mm, and those between the image centers of the Truebeam and SyncTraX FX4 were <0.2 mm respectively. The discrepancies for each coordinate among the four presets were <0.1 mm except for the AP direction (up to 0.16 mm) between the image centers of the Truebeam and the SyncTraX FX4.

**Table 2:** Discrepancy of the coordinates between the image/radiation center of TrueBeam and the center of SyncTraX FX4

	Image center of TrueBeam and image center of SyncTraX FX4			Radiation center of TrueBeam and image center of SyncTraX FX4		
	AP	SI	LR	AP	SI	LR
Preset #1	−0.11	0.03	−0.06	0.40	0.32	0.04
Preset #2	0.05	0.08	−0.10	0.39	0.26	0.01
Preset #3	0.00	0.00	−0.10	0.38	0.33	−0.05
Preset #4	−0.03	0.07	−0.04	0.37	0.22	0.06

All data are in mm. AP: anterior–posterior; SI: superior–inferior; LR: left–right.

### 3.C Comparison of the localization accuracy of CBCT and the SyncTraX FX4

**Figure 8:**
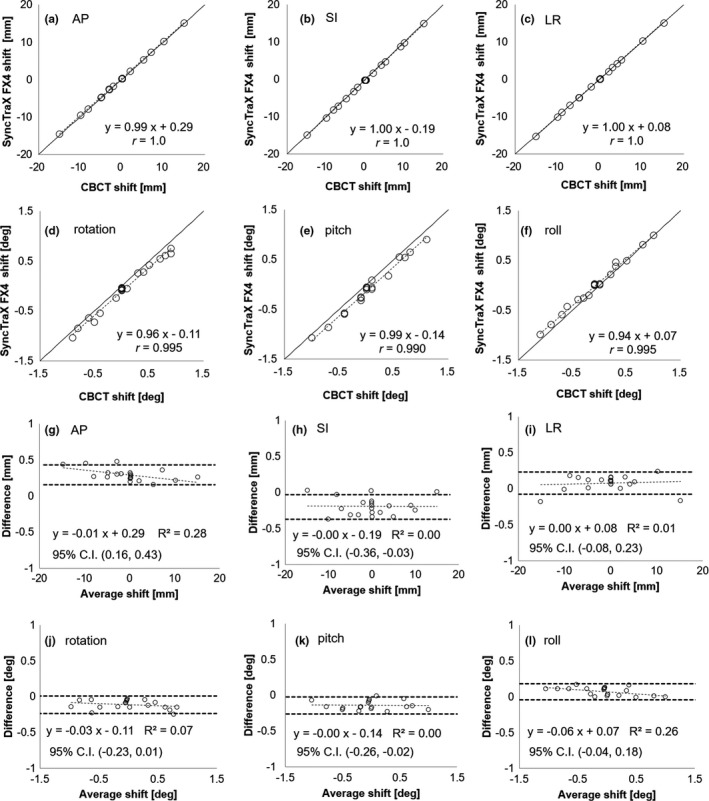
(a)–(f) Two‐dimensional correlation plots. (g)–(l) Bland‐Altman error analysis between CBCT and preset #3 in the SyncTraX FX4 system.

The mean differences, SD, and RMS values between the CBCT and SyncTraX FX4‐based shifts for preset #3 were (0.29, 0.084, 0.082 mm) for AP, (−0.19, 0.13, 0.12 mm) for SI, (0.076, 0.11, 0.10 mm) for LR, (−0.11°, 0.066°, 0.064°) for rotation, (−0.14°, 0.064°, 0.063°) for pitch, and (0.072°, 0.058°, 0.056°) for roll. The results obtained with the other three presets are summarized in Table 3. The maximum values of the differences among the presets were 0.09 mm for the translational shifts and 0.28° for the rotational shifts. Figure 8 shows the two‐dimensional correlations between the CBCT shift and the SyncTraX FX4‐based shift for preset #3. Pearson's correlation coefficients (*r*) were 1.0 for AP, 1.0 for SI, 1.0 for LR, 0.995 for rotation, 0.990 for pitch, and 0.995 for roll.

**Table 3:** Mean value, standard deviation, and RMS between the CBCT and SyncTraX FX4

	AP mm	SI mm	LR mm	Rotation deg	Pitch deg	Roll deg
Mean						
Preset #1	0.32	−0.25	0.050	−0.25	−0.17	0.074
Preset #2	0.29	−0.16	0.12	0.033	−0.18	0.090
Preset #3	0.29	−0.19	0.076	−0.11	−0.14	0.072
Preset #4	0.30	−0.21	0.11	−0.16	−0.15	0.063
SD						
Preset #1	0.067	0.14	0.13	0.11	0.10	0.060
Preset #2	0.080	0.12	0.10	0.059	0.047	0.053
Preset #3	0.084	0.13	0.11	0.066	0.064	0.058
Preset #4	0.083	0.12	0.12	0.076	0.083	0.071
RMS						
Preset #1	0.065	0.13	0.12	0.10	0.10	0.059
Preset #2	0.078	0.12	0.10	0.057	0.046	0.052
Preset #3	0.082	0.12	0.10	0.064	0.063	0.056
Preset #4	0.081	0.12	0.11	0.074	0.081	0.069

CBCT: cone‐beam computed tomography; RMS: root‐mean‐square; SD: standard deviation.

In the Bland‐Altman error analysis, the 95% confidence interval was (0.16, 0.43 mm) for AP, (−0.36, −0.03 mm) for SI, (−0.08, 0.23 mm) for LR, (−0.23°, 0.01°) for rotation, (−0.26°, −0.02°) for pitch, and (−0.04°, 0.23°) for roll, indicating no fixed bias in LR, rotation, or the roll direction between the CBCT and SyncTraX FX4. Similar correlations were observed in the other presets.

### 3.D Radiation dose of the SyncTraX FX4

**Figure 9:**
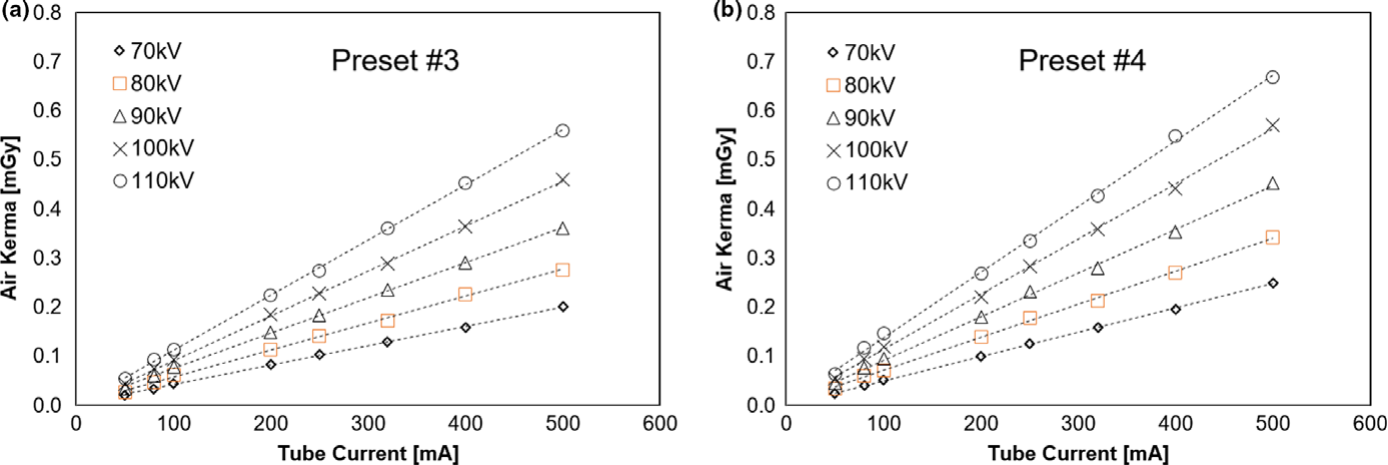
Relationship between the tube current and air kerma in (a) Preset #3 and (b) Preset #4.

The relationship between the tube current and air kerma for each voltage of the x‐ray tube for presets #3 and #4 is illustrated in Fig. 9. In both presets, the air kerma increased with the increase in the tube current and tube voltage. The mean air kerma values at 100 mA for preset #3 were 0.044 ± 0.00025 mGy for 70 kV, 0.079 ± 0.00031 mGy for 80 kV, 0.062 ± 0.00017 mGy for 90 kV, 0.093 ± 0.00035 mGy for 100 kV, and 0.11 ± 0.00075 mGy for 110 kV. In case of preset #4, the mean air kerma values at 100 mA were 0.052 ± 0.00036 mGy for 70 kV, 0.071 ± 0.00018 mGy for 80 kV, 0.095 ± 0.00053 mGy for 90 kV, 0.12 ± 0.00052 mGy for 100 kV, and 0.015 ± 0.00035 mGy for 110 kV. At any tube current and tube voltage, the air kerma values for preset #4 were larger than those for preset #3.

### 3.E End‐to‐end test

**Figure 10:**
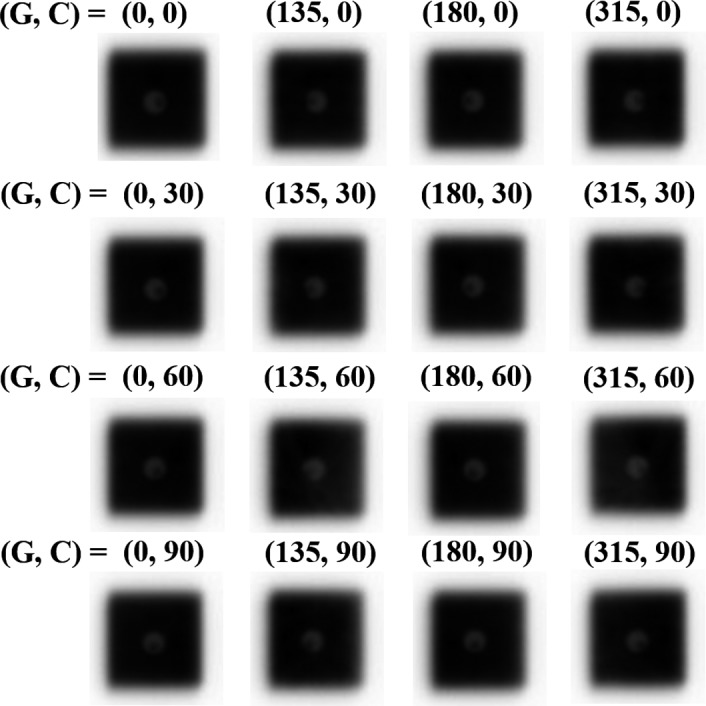
The images from the end‐to‐end test. The numbers followed by “G” and “C” are the gantry and couch angles respectively.

The results in the end‐to‐end test acquired by EPID for preset #3 are shown in Fig. 10. For 16 combinations of gantry and couch angles, the median value of the offset between the center of the marker and the image center was 0.31 mm (range, 0.14–0.49 mm). No angular dependence of the offsets on the gantry or couch rotations was observed. The same tendency was observed for each preset, as shown in Table 4.

**Table 4:** Median value of the offset between the center of the marker and the image center for each preset in the end‐to‐end test

	Median, mm (range)
Preset #1	0.35 (0.14–0.57)
Preset #2	0.31 (0.27–0.49)
Preset #3	0.31 (0.14–0.49)
Preset #4	0.31 (0.14–0.57)

## 4 DISCUSSION

This report is the first of an evaluation of the accuracy of the SyncTraX FX4 system via a clinical commissioning for intracranial SRT. The results of our analyses of the present commissioning series demonstrated that the performance of the SyncTraX FX4 system is sufficient for intracranial SRT.

Regarding the coincidence between the imaging center and radiation center for SRT, Kim et al. carried out similar tests at the clinical commissioning of a Novalis Tx linear accelerator, and they reported that the offsets were −0.7 ± 0.2 mm, −0.6 ± 0.2 mm, and 0.0 ± 0.2 mm in AP, SI, and LR directions respectively.^5^ In an assessment of spatial uncertainties in radiotherapy with a Novalis system, Hayashi et al. observed that the deviation of the center offsets between the ExacTrac x‐ray system and the radiation center were within 0.5 mm. In the present study, it became clear that the center offset between the imaging center of the SyncTraX FX4 system and the radiation center of the TrueBeam was sufficiently small (<0.4 mm) in all presets. These findings indicate that the SyncTraX FX4 provides a competent performance as an IGRT system.

Regarding positional accuracy in radiotherapy for intracranial tumors, several studies compared the positioning accuracy of the CBCT and a floor‐mounted IGRT system.^5^, ^6^, ^9^, ^10^ Oh et al. evaluated the positional accuracy of CBCT and an ExacTrac x‐ray system in the analysis of 107 patients for intracranial stereotactic radiosurgery (SRS), and they reported that the RMS values of the difference between the CBCT and ExacTrac x‐ray system were <1.01 mm and <0.82° for on‐line matching.^9^ They concluded that although these differences were minor, they should not be ignored. In the study by Ma et al. comparing the positioning accuracy in a head phantom and 18 patients with intracranial tumors, the RMS values of the differences between CBCT and an ExacTrac x‐ray system were <0.5 mm, <0.2° for the phantom, and <1.5 mm, <1.0° for the patients.^6^ They also noted that the impact of rotation on the differences was minor but not negligible. The RMS values of the differences in the present study were smaller than those of the Ma et al. study (<0.13 mm, <0.10°), and we thus consider the present results acceptable for a clinical commissioning. However, as shown in Fig. 8, some systematic errors were discovered in the AP, SI and pitch directions, and it is therefore necessary to pay close attention to the possibility of such errors in clinical use.

The radiation dose for the SyncTraX FX4 system was 0.66 mGy at the highest imaging condition. This result was within the range of radiation doses (0.1–2.0 mGy) to the kV x‐ray images in the AAPM TG‐75 report^19^ and thus a clinically acceptable value. As recommended by the AAPM TG‐180 report,^20^ further clinical investigations are necessary from the viewpoint of positional accuracy and radiation dose.

The end‐to‐end test in the present study revealed that the positional accuracy (<1 mm) required for intracranial SRT was sufficiently satisfied, and gantry and couch angle dependencies did not occur. These results indicated that it is possible to deliver various noncoplanar beams with the same accuracy, and clinical significance is thus available with the SyncTraX FX4 system with respect to the positional verification.

Each test in the present study was conducted in accordance with the AAPM TG‐142 report. It is important to evaluate both the trends in image quality and the coincidence between the imaging coordinate and that of the treatment systems for monthly quality assurance (QA), and the radiation dose for the annual QA. The TG‐142 report does not provide a recommendation about how frequently the degree of coordinate coincidence between two verification systems (such as CBCT and the SyncTraX FX4 system) should be measured, but we recommended that such a measurement should be conducted every 6 months.

In the present study, we focused on intracranial SRT and conducted a clinical commissioning of the SyncTraX FX4 system. However, each of the FPDs of the SyncTraX FX4 system has an effective field of view of 15 × 15 cm at the isocenter, and then can be used to perform positional verification for the chest and pelvic regions. The commissioning process described herein will be a reference for planning and executing a commissioning at each institution regardless of tumor sites.

## 5 CONCLUSIONS

We evaluated the accuracy of the SyncTraX FX4 system through a clinical commissioning for intracranial SRT. The results of our analyses demonstrated that intracranial SRT using this system can be realized with clinically acceptable accuracy.

## CONFLICT OF INTEREST

The authors have no conflicts of interest to declare.
